# CD44 Plays a Functional Role in *Helicobacter pylori*-induced Epithelial Cell Proliferation

**DOI:** 10.1371/journal.ppat.1004663

**Published:** 2015-02-06

**Authors:** Nina Bertaux-Skeirik, Rui Feng, Michael A. Schumacher, Jing Li, Maxime M. Mahe, Amy C. Engevik, Jose E. Javier, Richard M. Peek Jr, Karen Ottemann, Veronique Orian-Rousseau, Gregory P. Boivin, Michael A. Helmrath, Yana Zavros

**Affiliations:** 1 Department of Molecular and Cellular Physiology, University of Cincinnati, Cincinnati, Ohio, United States of America; 2 Department of Surgery, Division of Pediatric Surgery, Cincinnati Children’s Hospital Medical Center, Cincinnati, Ohio, United States of America; 3 Cancer Biology, Vanderbilt University, Nashville, Tennessee, United States of America; 4 Department of Microbiology and Environmental Toxicology, University of California at Santa Cruz, Santa Cruz, California, United States of America; 5 Karlsruhe Institute of Technology, Institute for Toxicology and Genetics, Hermann von Helmholtzplatz, Germany; 6 Department of Pathology Wright State University, Health Sciences, Dayton, Ohio, United States of America; 7 Veterans Affairs Medical Center, Cincinnati, Ohio, United States of America; University of Illinois, UNITED STATES

## Abstract

The cytotoxin-associated gene (Cag) pathogenicity island is a strain-specific constituent of *Helicobacter pylori* (*H. pylori*) that augments cancer risk. CagA translocates into the cytoplasm where it stimulates cell signaling through the interaction with tyrosine kinase c-Met receptor, leading cellular proliferation. Identified as a potential gastric stem cell marker, cluster-of-differentiation (CD) CD44 also acts as a co-receptor for c-Met, but whether it plays a functional role in *H. pylori*-induced epithelial proliferation is unknown. We tested the hypothesis that CD44 plays a functional role in *H. pylori*-induced epithelial cell proliferation. To assay changes in gastric epithelial cell proliferation in relation to the direct interaction with *H. pylori*, human- and mouse-derived gastric organoids were infected with the G27 *H. pylori* strain or a mutant G27 strain bearing cagA deletion (∆*CagA::cat*). Epithelial proliferation was quantified by EdU immunostaining. Phosphorylation of c-Met was analyzed by immunoprecipitation followed by Western blot analysis for expression of CD44 and CagA. *H. pylori* infection of both mouse- and human-derived gastric organoids induced epithelial proliferation that correlated with c-Met phosphorylation. CagA and CD44 co-immunoprecipitated with phosphorylated c-Met. The formation of this complex did not occur in organoids infected with ∆*CagA::cat*. Epithelial proliferation in response to *H. pylori* infection was lost in infected organoids derived from CD44-deficient mouse stomachs. Human-derived fundic gastric organoids exhibited an induction in proliferation when infected with *H. pylori*that was not seen in organoids pre-treated with a peptide inhibitor specific to CD44. In the well-established Mongolian gerbil model of gastric cancer, animals treated with CD44 peptide inhibitor Pep1, resulted in the inhibition of *H. pylori*-induced proliferation and associated atrophic gastritis. The current study reports a unique approach to study *H. pylori* interaction with the human gastric epithelium. Here, we show that CD44 plays a functional role in *H. pylori*-induced epithelial cell proliferation.

## Author Summary

Chronic gastric inflammation, typically caused by *Helicobacter pylori* (*H*. *pylori*), is the most consistent lesion leading to cancer. During a well-choreographed interaction between *H*. *pylori* and the host, the progression from chronic inflammation to cancer involves gastric epithelial changes with evidence of hyperproliferation. Our knowledge of *H*. *pylori* pathogenesis is predominantly based on data generated from gastric cancer cell lines or animal models of inflammation. We report the development and use of a novel model of primary human and mouse cultured gastric epithelial cells that are organized into three-dimensional spheroid units containing a lumen, known as gastric organoids. To assay changes in gastric epithelial cell proliferation in relation to the direct interaction with *H*. *pylori*, human- and mouse-derived gastric organoids were infected with the bacteria. Cluster-of-differentiation gene (CD44) is a transmembrane receptor responsible for epithelial cell proliferation. We show that CD44 plays a functional role in *H*. *pylori*-induced proliferation. In a Mongolian gerbil animal model of *H*. *pylori*-induced gastric cancer, we show that inhibiting CD44 blocks epithelial proliferation and subsequently cancer progression in response to bacterial infection. Thus our study provides new insights into the role of CD44 in *H*. *pylori*-induced hyperproliferation and progression of gastric disease.

## Introduction

The major cause of chronic inflammation in the stomach is *Helicobacter pylori (H*. *pylori)* [1], and it is widely accepted that chronic inflammation is a trigger for the development of gastric cancer [2]. The severity and localization of the inflammation that results from *H*. *pylori* infection is believed to dictate the pathological consequence of disease. Individuals most at risk of developing gastric cancer are those in whom the bacteria colonize the corpus (or fundus) of the stomach, when acid secretion is impaired. The subsequent development of severe inflammation in the gastric fundus leads to atrophy of the acid-secreting parietal cells and subsequently further hypochlorhydria, metaplasia and carcinoma [3,4,5]. Given that individuals most at risk of developing gastric cancer are those in whom the bacteria colonize the corpus [3,4,5], the current research is focused on the use of human- and mouse-derived fundic gastric epithelium, cultured as 3-dimensional structures called gastrointestinal organoids, for the study of *H*. *pylori* pathogenesis.

The cytotoxin-associated gene (cag) pathogenicity island is a strain-specific constituent of *H*. *pylori* that augments cancer risk [6]. The cag pathogenicity island encodes a type IV secretion system that is a multimolecular complex that mediates the translocation of bacterial factors into the host cell [6,7]. Upon delivery into the host cells by the type IV cag secretion system, CagA translocates into the host cell cytoplasm where it can stimulate cell signaling through interaction with several host proteins [6,8,9] including the tyrosine kinase c-Met receptor [10,11,12]. CagA exerts effects within host cells that mediate carcinogenesis, including aberrant activation of phosphatidylinositol 3-phosphate kinase (PI3K) and β catenin, disruption of apical-junctional complexes, and loss of cellular polarity [13,14,15].

Another host molecule that may influence carcinogenesis in conjunction with *H*. *pylori* and CagA is the cluster-of-differentiation (CD) CD44 cell surface receptor for hyaluronate [16]. CD44 is a cell surface adhesion molecule, expressed on a variety of cells including gastric epithelial cells, that has recently been identified as a gastric cancer stem cell marker, whereby cells expressing CD44 have been shown to possess the properties of gastric cancer stem cells [17]. CD44 variant isoforms, in particular CD44v6, was identified as a marker for invasive intramucosal carcinoma and premalignant lesions [18]. Suzuki *et al*. [19] demonstrated that CagA CM motifs interact with Met leading to sustained PI3K-AKT signaling in response to *H*. *pylori* resulting in cellular proliferation. Notably, the isoform containing exon v6 (CD44v6) acts as the coreceptor for c-Met, most probably, through binding of c-Met ligand hepatocyte growth factor (HGF) [20,21]. The coreceptor function of CD44v6 for c-Met is of particular interest given that studies pinpoint CD44v6 as a marker of early invasive intramucosal gastric carcinoma [18]. Whether CD44v6 acts as a coreceptor for the function of c-Met in response to *H*. *pylori* infection is unknown.

Our current knowledge of *H*. *pylori* pathogenesis is largely based on data generated from gastric cancer cell lines or *in vivo* animal models of inflammation. Thus, despite extensive evidence demonstrating that *H*. *pylori* induces gastric epithelial changes, the direct impact of the bacterium on the normal epithelium is unclear. Culture of primary human- and mouse-derived gastric stem cells as 3-dimensional structures called gastrointestinal organoids are a rapidly emerging approach to study gastrointestinal development, physiology, stem cell biology and disease [22,23,24,25,26,27,28,29]. Troy-positive cells are expressed at the corpus gland base in a subset of differentiated chief cells [23]. Stange *et al*. [23] demonstrate that Troy-positive chief cells may be used to generate long-lived gastric organoids, but *in vitro* these cultures are differentiated toward the mucus-producing cell lineages of the neck and pit regions. The Troy-derived organoids are distinct from the cultures that we derive from whole dissociated glands reported here such that we have devised a method to maintain all the major cell lineages of the fundus [22,28]. In this investigation, we used our method of mouse-derived gastric organoid cultures as an approach to assay changes in gastric epithelial cell proliferation in relation to the direct interaction with *H*. *pylori* [22,24,29]. To study the functional role of CD44 in the context of human epithelial tissue, we developed a protocol for culturing human-derived gastric organoids. We developed cultures of human-derived fundic gastric organoids independent of the recent report by the Clevers group demonstrating the establishment of a similar culture model for the study of *H*. *pylori* pathogenesis [25]. Despite the extensive use of these culture systems for the study of stem cell biology and gastrointestinal development [22,23,24,25,26,27,29], the degree to which these cultures reflect the physiology of native tissue has been reported by our laboratory alone [28]. Here we extend our current knowledge of *H*. *pylori* pathogenesis by identifying the signaling mechanism by which bacterial infection induces proliferation in the gastric epithelium. While it is known that c-Met is an important CD44 partner in proliferation, this is the first report that this association occurs in response to *H*. *pylori* infection. We find that CD44 plays a functional role in *H*. *pylori-*induced proliferation both *in vitro* and *in vivo*.

## Results

### CD44 mediates *H*. *pylori*-induced proliferation

**Fig 1:**
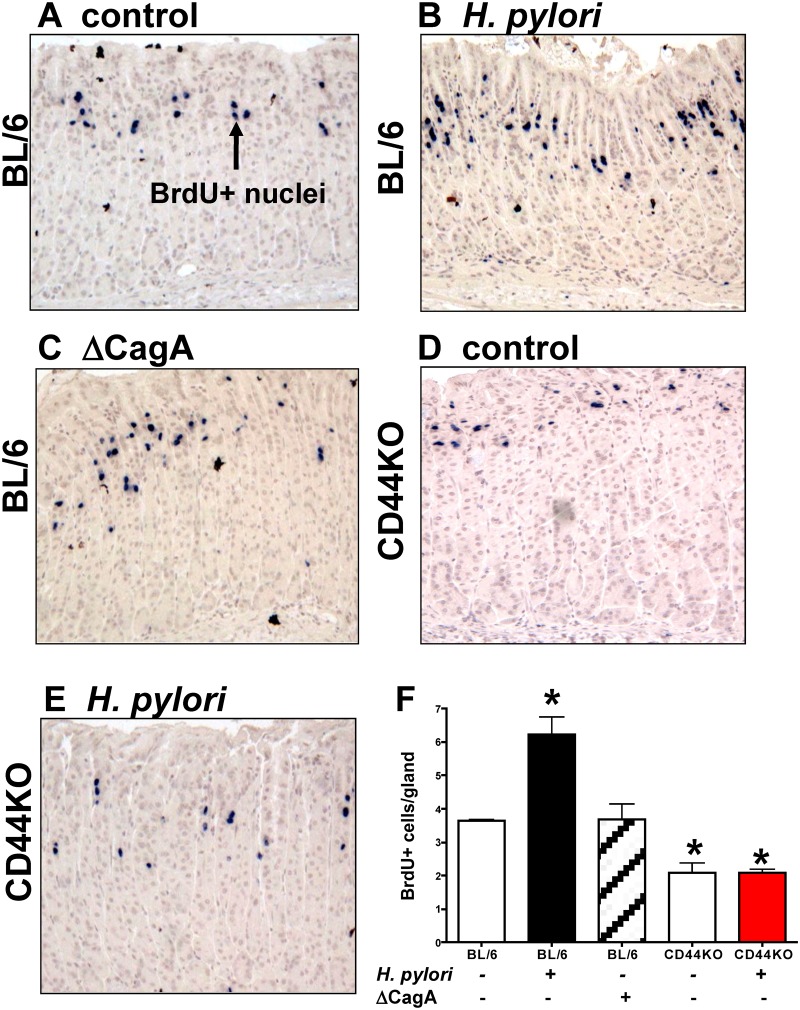
Epithelial proliferation in response to *H*. *pylori* infection of C57BL/6 (BL/6) and CD44 deficient (CD44KO) mice. Stomach sections collected from (**A**) uninfected BL/6 control, (**B**) *H*. *pylori* infected BL/6, (**C**) ∆CagA *H*. *pylori* strain infected BL/6, and (**D**) uninfected and (**E**) *H*. *pylori* infected CD44KO mice were immunostained for BrdU incorporation (blue nuclei). (**F**) Quantification of BrdU+ cells/gland in each group. *P<0.05 compared to controls, n = 6 mice per group.

To determine if CD44 plays a functional role in *H*. *pylori*-induced proliferation, C57BL/6 (BL/6) control and CD44 deficient mice were infected with mouse adapted LSH100 *H*. *pylori* strain for 4 weeks. The LSH100 mouse-adapted strain [30] is a descendant of the clinical isolate G27 [31]. We chose the LSH100 *H*. *pylori* to study the mechanism of bacterial-induced proliferation *in vivo* because this particular strain efficiently expresses virulence factor CagA [14,31,32,33]. The proliferating cells were measured by BrdU incorporation (Fig. 1). There was a significant increase of BrdU positive cells per gland in *H*. *pylori* LSH100 strain infected mice (6.23 + 0.53 BrdU+ cells/gland, Fig. 1B, E) compared to Brucella broth control mice (3.64 + 0.04 BrdU+ cells/gland, Fig. 1A, E). Infection of BL/6 mice with LSH100 strain bearing a CagA deletion (∆*CagA*) lacked the significant increase in proliferation (3.68 + 0.46 BrdU+ cells/gland, Fig. 1C, E). Importantly, CD44 deficient mice infected with *H*. *pylori* did not exhibit an increase in proliferation (2.09 + 0.11 BrdU+ cells/gland, Fig. 1D, E) when compared to the Brucella broth uninfected control CD44KO mouse group (2.10 + 0.29 BrdU+ cells/gland, Fig. 1E). These data show that CD44 mediates CagA dependent *H*. *pylori-*induced proliferation.

### *H*. *pylori* injection and viability in gastric organoids

**Fig 2:**
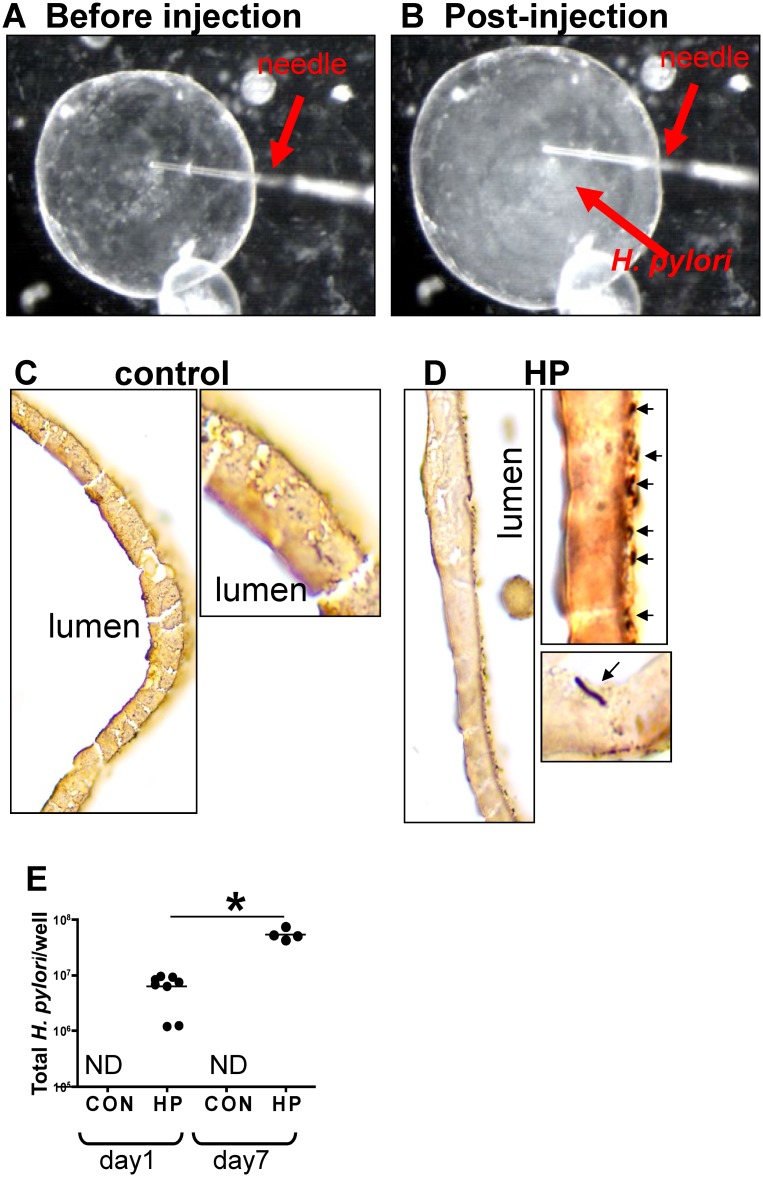
H. pylori microinjection and colonization of mFGOs. Organoids were microinjected with *H*. *pylori* shown are mFGOs (**A**) before and (**B**) after injection. Arrow indicates injection needle. ‘Cloud’ of *H*. *pylori* observed after injection is shown. Twenty-four hours after luminal microinjection, *H*. *pylori* (HP) attach to the luminal surface demonstrated by Warthin-Starry stain of (**C**) control and (**D**) HP infected organoids. (**E**) Quantitative cultures of HP of control and HP infected organoids. *P<0.05 compared to bacterial numbers at day 1 after infection.

To identify the direct impact of *H*. *pylori* on the host gastric epithelium, we employed the use of a mouse-derived gastric organoid culture system. We have previously described a system for culturing gastric organoids derived from mouse fundic tissue, in which the fundic organoids are embedded in Matrigel, provided gastric organoid growth media, and co-cultured in transwell plates with immortalized stomach mesenchymal cells (ISMCs) [22,28]. Organoids were microinjected with *H*. *pylori* strain G27. *H*. *pylori* strain *G27*, originally isolated from an endoscopy patient from Grosseto Hospital (Tuscany, Italy) [31], is readily transformable and therefore amenable to gene disruption [34]. Of relevance to the current study, strain G27 efficiently delivers the translocated virulence factor CagA to cells in culture [14,31,32,33]. Therefore, we chose G27 to study the mechanism of *H*. *pylori-*induced proliferation using a strain that efficiently expresses virulence factor CagA. Organoids were microinjected with *H*. *pylori* G27 strain (Fig. 2A), and bacterial adhesion was confirmed by Warthin-Starry stain (Fig. 2C, D) and culture (Fig. 2E). Quantitative cultures showed a significant increase in the bacteria cultured from organoids infected for 7 days compared to 24 hours and thus confirming bacterial viability within the cultures (Fig. 2E). Therefore, the fundic organoids provided a method by which bacterial-host cell interactions may be studied in the context of an intact normal gastric epithelium *in vitro*.

### CD44 mediates *H*. *pylori*-induced epithelial proliferation in mouse-derived fundic gastric organoids (mFGOs)

**Fig 3:**
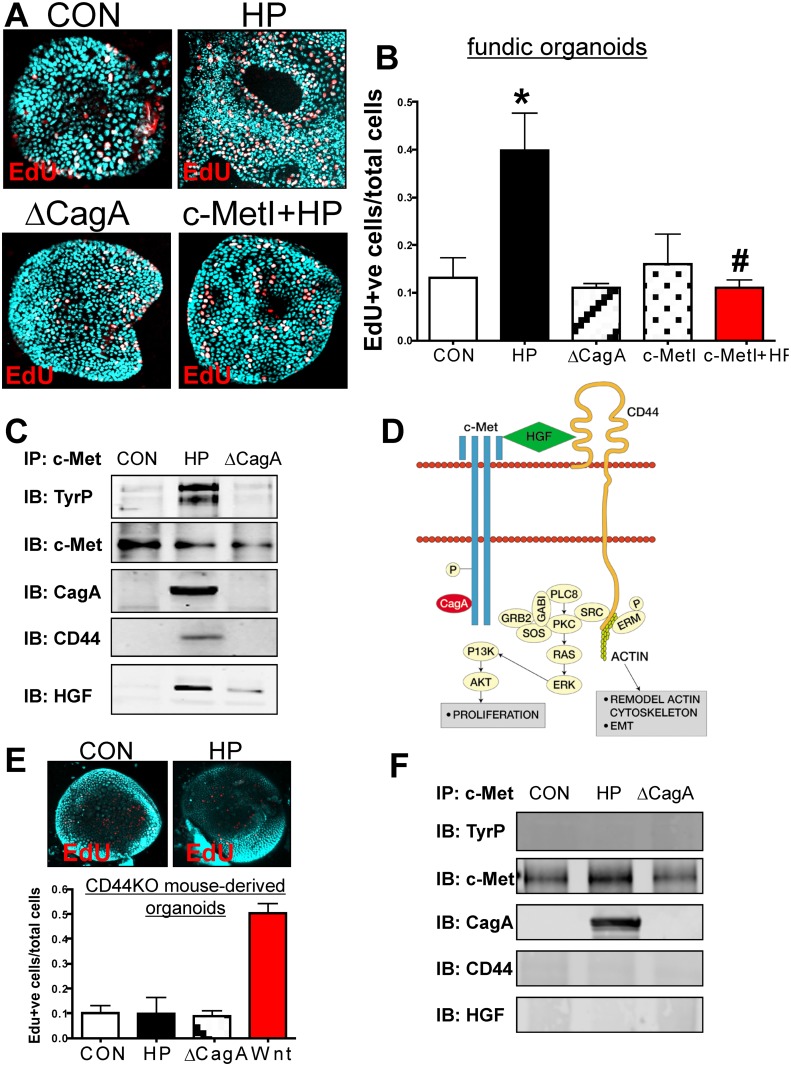
Changes in proliferation in *H*. *pylori* infected mFGOs. (**A**) Immunofluorescence of EdU+ cells in control (CON), *H*. *pylori* (HP) and HP ∆CagA infected and HP infected fundic organoids pretreated with c-Met inhibitor (c-MetI+HP). (**B**) Quantification of EdU+ cells/total cells. *P<0.05 compared to CON group, n = 6 individual organoid preps per group. (**C**) Protein lysates were prepared from mFGOs that were uninfected (CON), HP or ∆cagA infected for 24 hours. Lysates were immunoprecipitated using an anti-c-Met antibody and immunoblotted for TyrP, c-Met, CagA, CD44 and HGF. (**D**) Proposed model of the CD44 co-receptor function in response to *H*. *pylori*. (**E**) Immunofluorescence of EdU+ cells in CON, and HP infected organoids derived from CD44KO mouse fundus. Quantification of EdU+ cells in CON, HP and or ∆CagA infected and Wnt agaonist-treated (Wnt) organoids derived from CD44KO mouse fundus. *P<0.05 compared to CON group, n = 3 individual organoid preps per group. (**F**) Protein lysates were prepared from mFGOs derived from CD44-deficient mice that were uninfected (CON), HP or ∆CagA infected for 24 hours. Lysates were immunoprecipitated using an anti-c-Met antibody and immunoblotted for TyrP, c-Met, CagA, CD44 and HGF.

When the mFGOs were infected with *H*. *pylori* G27 strain, we observed a significant increase in epithelial cell proliferation in response to bacterial infection compared to the uninfected controls (Fig. 3A, B). The proliferative response to *H*. *pylori* was significantly blocked when organoids were pretreated with the c-Met inhibitor (c-MetI PF04217903 mesylate) (Fig. 3A, B). Organoids infected with the G27 *H*. *pylori* strain that expressed a deletion of CagA (∆CagA) did not differ from the controls with regards to proliferation (Fig. 3A, B). These data show that *H*. *pylori*-induced proliferation is mediated by activation of c-Met signaling as previously reported [19]. Therefore, to advance this current knowledge, we examined whether c-Met was associated with CD44. Lysates were prepared from uninfected organoids and organoids infected with either *H*. *pylori* (G27 strain) or ∆CagA and immunoprecipitated using an anti-c-Met antibody. Immunoprecipitates analyzed by Western blot using an anti-phosphotyrosine antibody showed an increase in phosphorylated c-Met in response to *H*. *pylori* (Fig. 3C). Consistent with published studies CagA coimmunoprecipitated with c-Met [19]. CD44 also coimmunoprecipitated with c-Met. C-Met is an important partner with CD44 in proliferation, but this is the first time that it has been reported that this association occurs in response to *H*. *pylori* infection. There is evidence suggesting CD44 binds to hepatocyte growth factor (HGF) and acts as a co-receptor by presenting HGF to c-Met and subsequently activating Met signaling [20,21] (Fig. 3D). In response to *H*. *pylori* infection, we show for the first time, HGF also coimmunoprecipitated with c-Met, and HGF expression was significantly upregulated in response to bacterial infection (Fig. 3C). Collectively, these data suggest that *H*. *pylori* induces the proliferation of the gastric epithelium by promoting an association between CagA, Met and CD44.

To test the functional role of CD44 in mouse-derived gastric organoids, we cultured organoids derived from mice that lacked the gene for CD44 (CD44KO). Organoids derived from the stomachs of CD44 deficient (CD44KO) mice did not proliferate in response to *H*. *pylori* infection (Fig. 3E). CD44KO mouse-derived organoids proliferated in response to a Wnt agonist, and thus showing that this lack of response was specific to *H*. *pylori* (Fig. 3E). To determine if CD44 was required for c-Met phosphorylation, lysates from uninfected control, *H*. *pylori* infected and ∆CagA infected organoids derived from stomachs of CD44-deficient mice were collected. In *H*. *pylori* infected CD44-deficient organoids c-Met was present but not phosphorylated (Fig. 3F). Importantly, in the absence of CD44, CagA co-immunoprecipitated with c-Met and is thus likely to form a complex with this receptor (Fig. 3F). In the absence of CD44, HGF was not detected in the c-Met immunoprecipitated protein complex (Fig. 3F). Collectively, these data further show that both CD44 and c-Met play a functional role in CagA dependent *H*. *pylori*-induced epithelial cell proliferation in mouse-derived fundic gastric organoids.

### *H*. *pylori* infection triggers an epithelial-to-mesenchymal transition in gastric organoids

**Fig 4:**
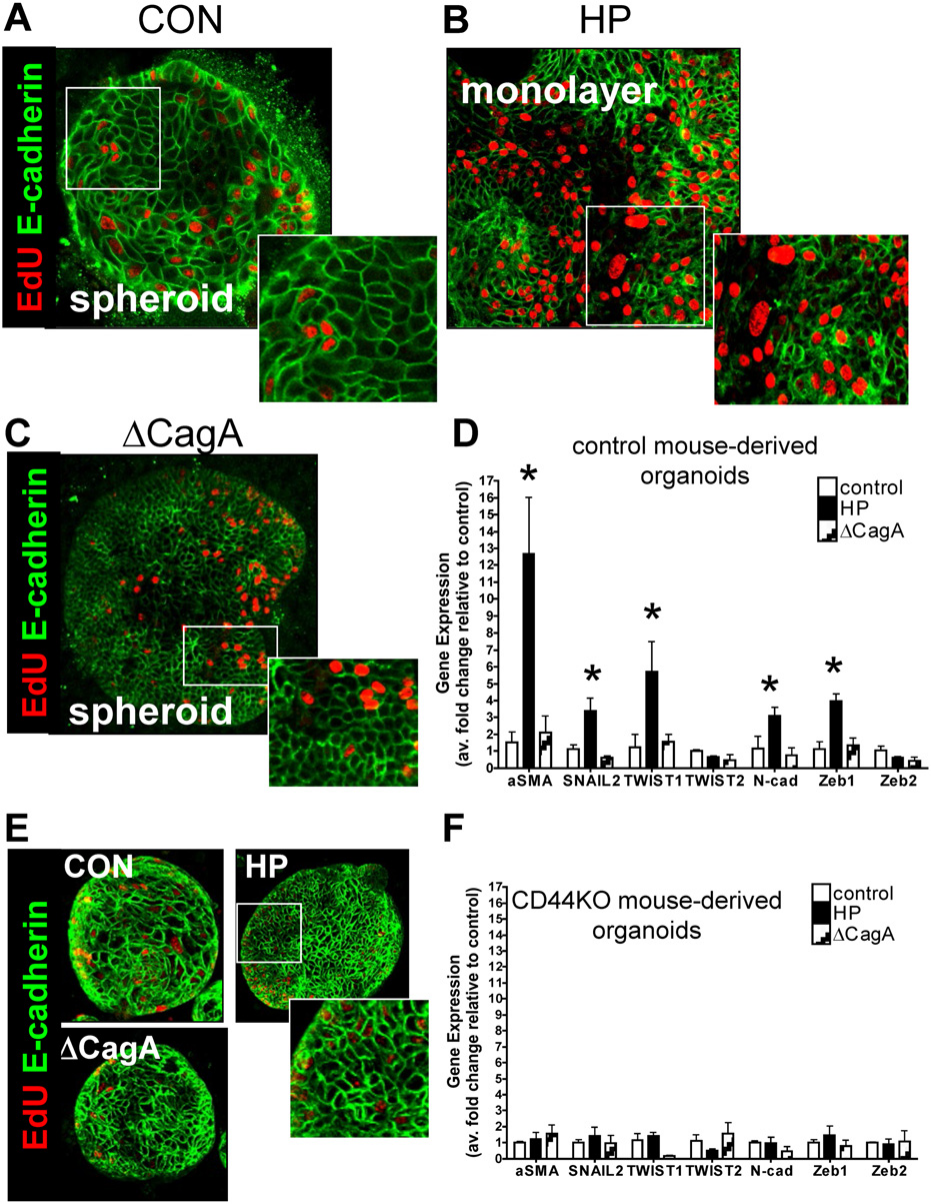
*H. pylori*-induced epithelial-to-mesenchymal transition (EMT). Immunofluorescence of EdU (red) and E-cadherin (green) using (**A**) uninfected control (CON), (**B**) *H*. *pylori* (HP) or (**C**) ∆CagA infected mFGOs. (**D**) EMT marker (αSMA, SNAIL, TWIST, N-cadherin and Zeb) expression measured by qRT-PCR using RNA isolated from mFGOs uninfected (control), *H*. *pylori* (HP) or ∆CagA infected. *P<0.05 compared to CON group, n = 4 individual experiments/group. (**E**) Immunofluorescence of EdU (red) and E-cadherin (green) using uninfected control (CON), *H*. *pylori* (HP) and ∆CagA infected mFGOs derived from stomachs of CD44-deficient mice. (**F**) (**D**) EMT marker (αSMA, SNAIL, TWIST, N-cadherin and Zeb) expression measured by qRT-PCR using RNA isolated from mFGOs derived from CD44-deficient mouse stomachs uninfected (control), *H*. *pylori* (HP) or ∆CagA infected.

In addition to increased epithelial cell proliferation, we observed striking morphological changes in response to *H*. *pylori* infection that were consistent with epithelial-to-mesenchymal transition (EMT) (Fig. 4). In uninfected control (CON) mouse-derived fundic gastric organoids, we observed clear membrane-expressed E-cadherin (Fig. 4A). However, in the *H*. *pylori* G27 strain infected organoids there was a disruption in membrane-expressed E-cadherin and transition of the spheroid morphology to a cell monolayer was observed (Fig. 4B). In control mouse-derived fundic gastric organoids infected with the ∆cagA strain, we also observed clear membrane-expressed E-cadherin (Fig. 4C). EMT was also documented by increased expression of markers that included alpha smooth muscle actin (αSMA), SNAIL2, TWIST1, N-cadherin and Zeb1 (Fig. 4D). Organoids derived from the stomachs of CD44KO mice were also microinjected with Brucella broth (uninfected control), *H*. *pylori* G27 strain or *H*. *pylori* ∆cagA strain. In all three groups membrane-expressed E-cadherin was observed (Fig. 4E). In addition changes in gene expression of αSMA, SNAIL2, TWIST1, N-cadherin and Zeb1 was not observed in infected CD44KO mouse-derived organoids (Fig. 4F). These data show that *H*. *pylori* induces an EMT phenotype in the mouse-derived fundic gastric organoids and that this response may be mediated by CD44.

### Development and characterization of human fundic gastric organoids (hFGOs)

**Fig 5:**
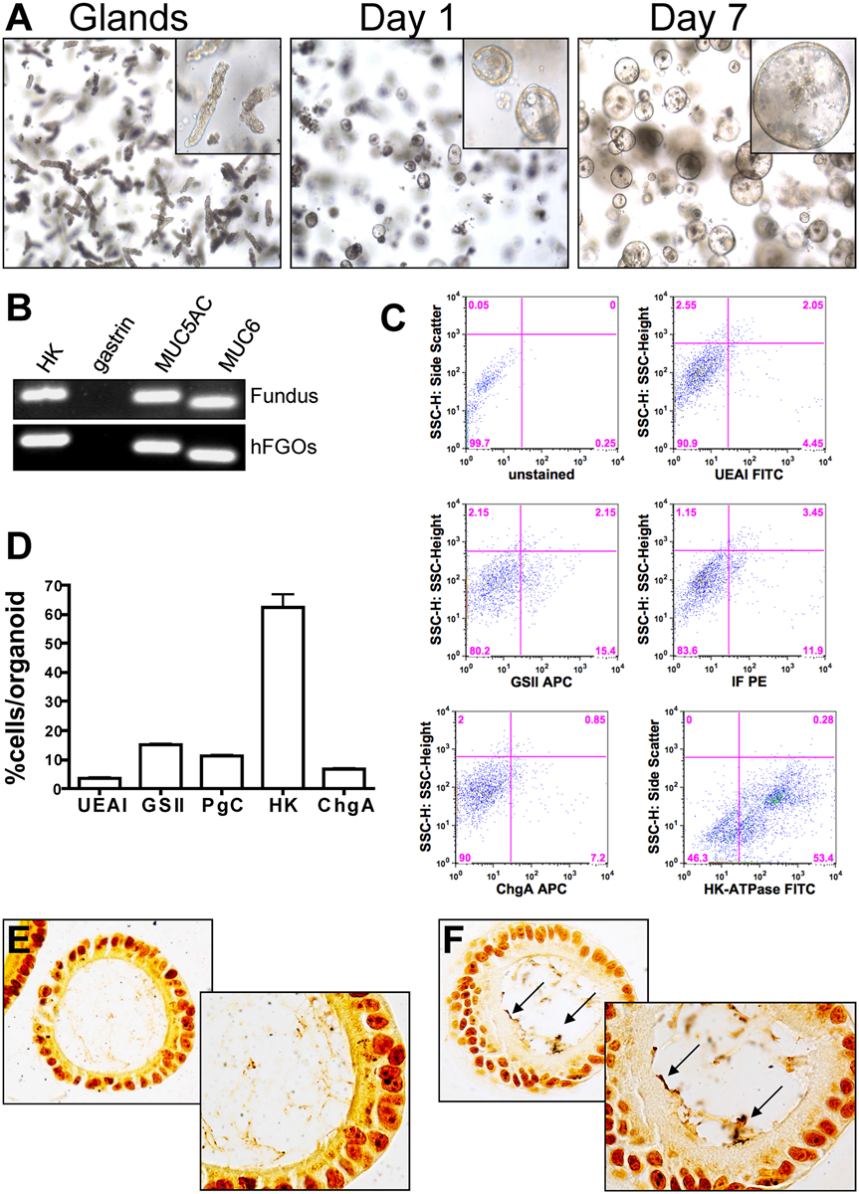
Development and characterization of hFGOs. (**A**) Growth of human fundic glands into 3-dimensional epithelial spheres at day 1 and day 7. (**B**) The hFGOs express markers normally found in human fundus including H^+^/K^+^ ATPase (HK), Muc5ac, and Muc6, but lack expression of the antral specific marker gastrin. (**C**) Flow cytometric analysis of lineage markers for human fundus including UEAI, GSII, pepsinogen C (PgC), Chromogranin A (ChgA), and H^+^/K^+^ ATPase (HK). (**D**) Quantification of flow cytometric data shown in (**C**). Warthin-Starry staining on (**E**) uninfected control and (**F**) *H*. *pylori* injected hFGOs.

To identify the role of CD44 as a mediator of *H*. *pylori*-induced proliferation in human epithelium, we developed the human-derived fundic gastric organoids (hFGOs). Gastric glands were enzymatically dissociated from the human fundic tissue embedded in Matrigel, provided gastric organoid growth media and cultured to form hFGOs epithelial spheres over 7 days (Fig. 5A). These 3 dimensional epithelial spheres contained markers specific for the fundic epithelium, including H^+^,K^+^ ATPase (HK), Muc5ac, and Muc6, but do not contain gastrin, a marker for the antral region of the stomach (Fig. 5B). Flow cytometric analysis showed that the majority of cells in the human organoids were positive for the marker for parietal cells, H^+^,K^+^ ATPase, as would be expected in oxyntic glands in the human stomach (Fig. 5C). Quantification of the flow cytometry histograms revealed that the hFGOs were comprised of approximately 5% UEAI+ cells (surface mucous pit cells), 15% GSII+ cells (mucous neck cells), 12% pepsinogen C (PgC)+ cells (chief cells), 7% chromogranin A+ cells (ChgA endocrine cells) and 60% H^+^,K^+^ ATPase+ cells (HK, parietal cells) (Fig. 5D). As detailed for the mouse-derived fundic gastric organoids, hFGOs were microinjected with *H*. *pylori* G27 strain and bacterial adhesion was confirmed by Warthin-Starry stain (Fig. 5E, F). Therefore, the hFGOs provided a method by which bacterial-host cell interactions were studied in the context of an intact normal human gastric epithelium *in vitro*.

### CD44 mediates *H*. *pylori-*induced proliferation in hFGOs

**Fig 6:**
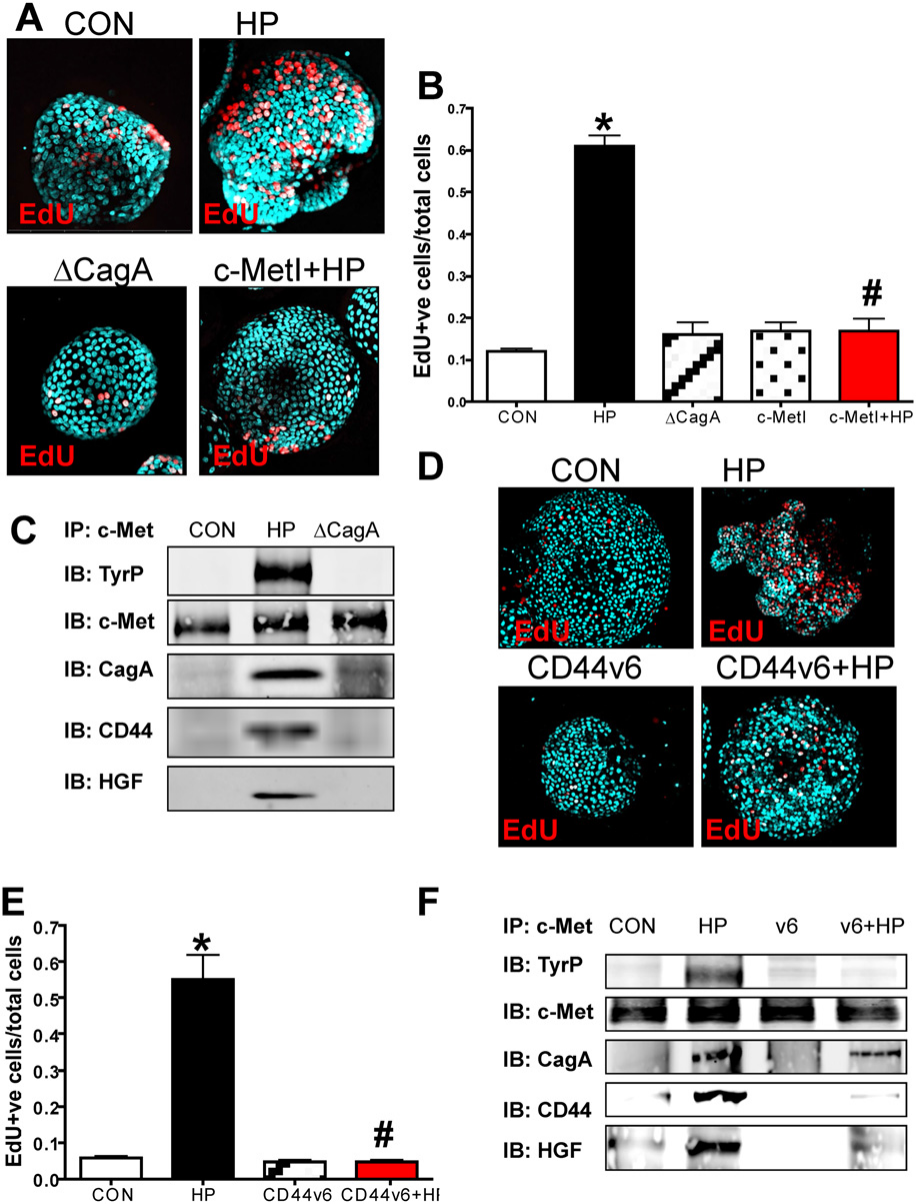
Changes in proliferation in *H*. *pylori* infected hFGOs. (**A**) Immunofluorescence of EdU+ cells in control (CON), *H*. *pylori* (HP) and HP ΔCagA infected and HP infected fundic organoids pretreated with c-Met inhibitor (c-MetI+HP). (**B**) Quantification of EdU+ cells/total cells. *P<0.05 compared to CON group, n = 6 individual organoid preps per group. (**C**) Protein lysates were prepared from hFGOs that were uninfected (CON), HP or ΔcagA infected for 24 hours. Lysates were immunoprecipitated using an anti-c-Met antibody and immunoblotted for TyrP, c-Met, CagA and CD44. (**D**) Immunofluorescence of EdU+ cells in control (CON), *H*. *pylori* (HP) infected, CD44v6 neutralizing antibody treated (CD44v6) and HP infected pretreated with CD44v6 neutralizing antibody (CD44v6+HP) hFGOs. (**E**) Quantification of EdU+ve cells/total cells. *P<0.05 compared to CON group, #P<0.05 compared to HP infected group, n = 4 individual organoid preps per group. (**F**) Protein lysates were prepared from hFGOs that were uninfected (CON), *H*. *pylori* (HP) infected, CD44v6 neutralizing antibody treated (CD44v6) and HP infected pretreated with CD44v6 neutralizing antibody (CD44v6+HP). Lysates were immunoprecipitated using an anti-c-Met antibody and immunoblotted for TyrP, c-Met, CagA and CD44.

To determine if the co-receptor role of CD44 and c-Met was intact in human tissue, hFGOs were infected with *H*. *pylori* G27 strain. Compared to the control treated (CON) hFGOs, infection with *H*. *pylori* triggered a significant induction in proliferating cells that was not seen in organoids injected with the ∆CagA mutant strain (Fig. 6A, B). Consistent with the response observed in the mouse-derived organoids, the proliferative response to *H*. *pylori* was significantly blocked when hFGOs were pretreated with the c-Met inhibitor (c-MetI PF04217903 mesylate) (Fig. 6A, B). As detailed for the mouse-derived fundic gastric organoids, lysates were prepared from uninfected and hFGOs infected with either *H*. *pylori* (G27 strain) or ∆CagA and immunoprecipitated using an anti-c-Met antibody. Immunoprecipitates analyzed by western blot using an anti-phosphotyrosine antibody showed an increase in phosphorylated c-Met in response to *H*. *pylori* (Fig. 6C). CagA, CD44 and HGF co-immunoprecipitated with c-Met. It is known that c-Met is an important partner for CD44 in proliferation, but this is the first report of the CD44/c-Met association occuring in response to *H*. *pylori* infection of the human gastric epithelium.

To test the function of CD44v6 in hFGOs, we used a neutralizing antibody that specifically targets human CD44v6. We found *H*. *pylori*-induced proliferation was blocked in hFGOs pre-treated with the CD44v6 neutralizing antibody for one hour prior to *H*. *pylori* injection (Fig. 6D, E). Treating hFGOs with the CD44v6 neutralizing antibody alone did not significantly change the baseline of proliferation seen in the control hFGOs (Fig. 6D, E). Consistent with data shown in Fig. 6C, immunoprecipitates analyzed by western blot showed an increase in phosphorylated c-Met in response to *H*. *pylori* and CagA, CD44 and HGF co-immunoprecipitated with c-Met (Fig. 6F). Interestingly, inhibition of CD44 binding to hyaluronic acid using the CD44v6 peptide inhibited the co-immunoprecipitation of CD44 and HGF with c-Met (Fig. 6F). These data indicate that CD44v6 plays a functional role in *H*. *pylori-*induced proliferation in hFGOs, and that c-Met may collaborate with CD44 in this proliferative response to *H*. *pylori* infection in human tissue.

### CD44 signaling mediates *H*. *pylori*-induced atrophic gastritis and hyperproliferation in the Mongolian gerbil model of gastric cancer

**Fig 7:**
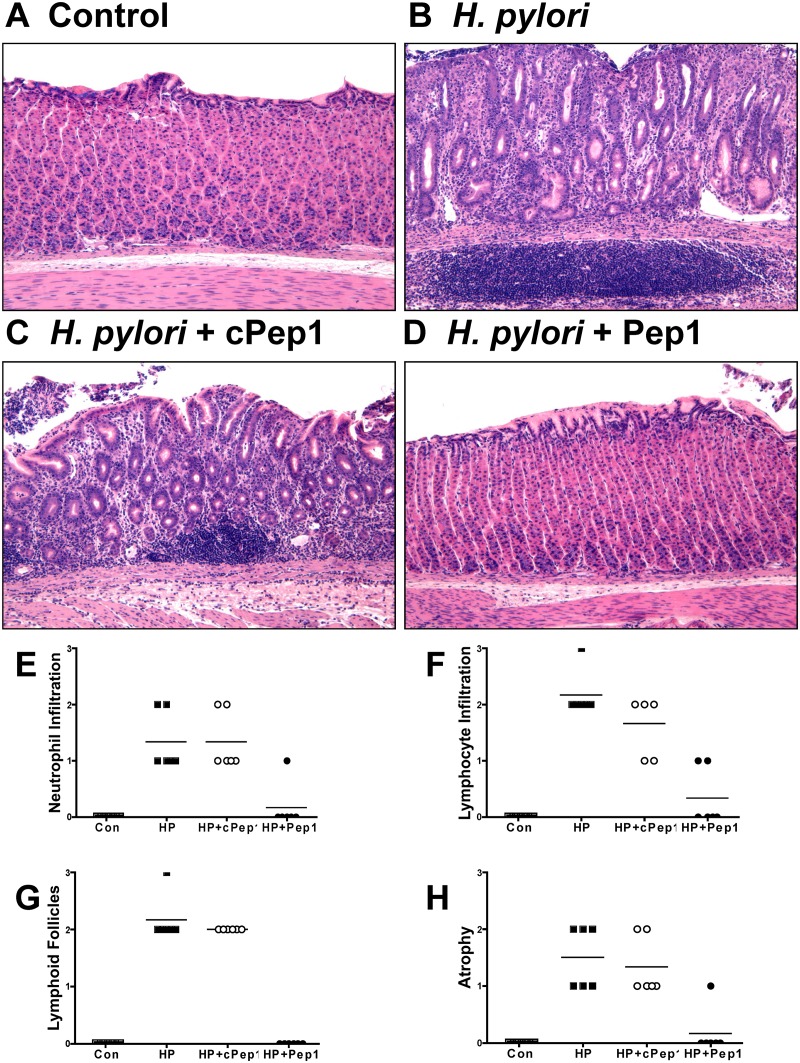
Inhibition of CD44 blocks the development of atrophic gastritis in *H*. *pylori* infected Mongolian gerbils. Mongolian gerbils were administered (**A**) *Brucella* broth (control), (**B**) *H*. *pylori* strain 7.13 alone, and in combination with either a (**C**) control peptide (cPep1), or (**D**) peptide targeting CD44 (Pep1). Gerbils were sacrificed after 6 weeks infection and H&E’s were scored using the updated Sydney classification for (**E**) neutrophil infiltration, (**F**) lymphocyte infiltration, (**G**) lymphoid follicles, and (**H**) atrophy.

We next investigated the role of CD44 in cancer progression using a well-established Mongolian gerbil model of gastric cancer [35,36]. We administered an inhibitory peptide, Pep1, that prevents binding of CD44 ligand hyaluronic acid (HA), thus blocking CD44 downstream signaling. Gerbils were infected with *H*. *pylori* strain 7.13, an *in vivo* adapted strain originally isolated from a gastric ulcer [37] and reported to reproducibly induce gastric cancer in Mongolian gerbils [13,37]. We found that gerbils infected with *H*. *pylori* strain 7.13, developed atrophic gastritis (Fig. 7B), as documented by neutrophil (Fig. 7E) and lymphocyte (Fig. 7F) infiltration, development of lymphoid follicles (Fig. 7G) and atrophy (Fig. 7H), 6 weeks of infection, compared to control (*Brucella* broth administered) gerbils (Fig. 7A, E-H). Gerbils that received Pep1 injections three times a week for the duration of the 6 week infection with *H*. *pylori*, did not exhibit the development of atrophic gastritis (Fig. 7D, E-H), compared to the gerbils who received *H*. *pylori* and the scrambled control peptide (cPep1) (Fig. 7C, E-H).

**Fig 8:**
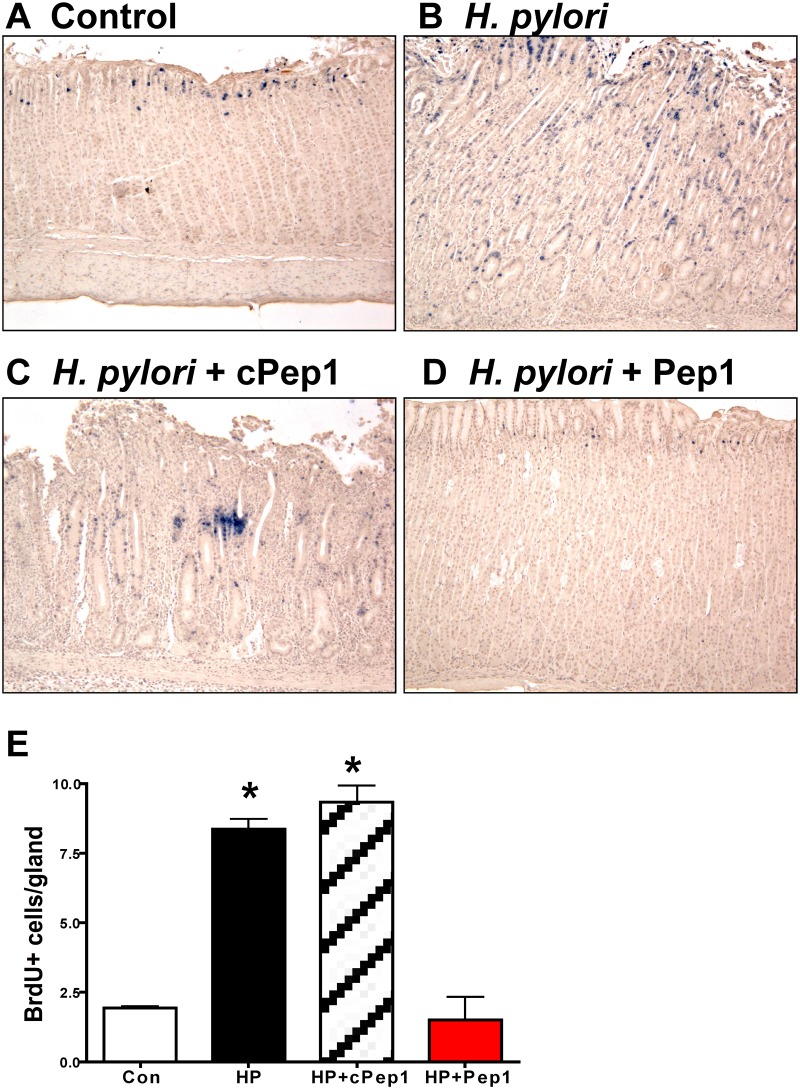
Epithelial proliferation in response to *H*. *pylori* infection in Mongolian gerbils. Stomach sections collected from Mongolian gerbils administered (**A**) *Brucella* broth (control), (**B**) *H*. *pylori* strain 7.13 alone, and in combination with either a (**C**) control peptide (cPep1), or (**D**) peptide targeting CD44 (Pep1) were immunostained for BrdU incorporation (blue nuclei). (**E**) Quantification of BrdU+ cells/gland in each group. *P<0.05 compared to controls, n = 6 gerbils per group.

Our studies show that CD44 plays a functional role in *H*. *pylori*-induced proliferation of mouse and human epithelial tissues *in vitro*. Thus, we next examined the proliferative state in response to *H*. *pylori* and Pep1 treatment in the Mongolian gerbils *in vivo*. Within 6 weeks of infection with *H*. *pylori* (7.13) there was a significant induction in the number of proliferating cells in the gastric epithelium (Fig. 8B, E) when compared to *Brucella* broth controls (Fig. 8A, E). To determine if CD44 signaling was required for this *H*. *pylori* induced proliferative response, we treated the *H*. *pylori* infected gerbils three times a week with Pep1, and found that the induction in proliferation was blocked (Fig. 8C, E). The gerbils infected with *H*. *pylori* and treated with the scrambled control peptide (cPep1) exhibited the expected induction in proliferation (Fig. 8D, E). Collectively, these data show that CD44 signaling mediates *H*. *pylori*-induced atrophic gastritis and hyperproliferation in the Mongolian gerbil model of gastric cancer.

## Discussion

Prolonged cell proliferation in the gastric mucosa is a precursor to the progression from chronic inflammation to gastric cancer in response to *H*. *pylori* infection [2]. However, the mechanism by which *H*. *pylori* induces epithelial cell proliferation is not well defined. Here we report the culture of primary human- and mouse-derived gastric epithelial cells as 3-dimensional structures called gastrointestinal organoids for the study of *H*. *pylori* pathogenesis. We utilized mouse-derived fundic gastric organoids (mFGOs), and for the first time we demonstrated that CD44 and c-Met form a complex with virulence factor CagA in response to *H*. *pylori* infection. To determine if this mechanism was present in human gastric epithelium, we developed a novel protocol for the culture of differentiated human-derived fundic gastric organoids in culture (hFGOs). In the hFGOs, we found that inhibiting CD44 splice variant 6, a specific marker for early invasive carcinoma [38], blocked *H*. *pylori*-induced proliferation. As in the mouse organoids, *H*. *pylori* infection triggered the formation of a complex containing CD44, c-Met, and CagA in the hFGOs. In addition, in the well-established Mongolian gerbil model of gastric cancer, animals treated with CD44 peptide inhibitor Pep1, resulted in the inhibition of *H*. *pylori-*induced proliferation and associated atrophic gastritis.

Our experiments report for the first time that CD44 acts as a mediator of *H*. *pylori*-induced proliferation both *in vitro* and *in vivo*. *In vivo*, we observed that CD44 regulates baseline proliferation whereby, as previously reported [39], basal rate of proliferation was approximately half that of control mice. However, *H*. *pylori* infection did not induce epithelial cell proliferation within the CD44KO mice, suggesting a functional role of CD44 as a mediator of bacterial-induced proliferation. Consistent with previous findings in gastric cancer cell lines [10], we also report that the proliferative response to *H*. *pylori* was CagA- and c-Met-dependent within the gastric epithelium. Western blot analysis using lysates collected from *H*. *pylori* infected mFGOs and hFGOs also showed that CagA coimmunoprecipitates with c-Met, as previously shown in human gastric cancer cells [19]. Although we report that *H*. *pylori*-induced proliferation was dependent on CD44 expression and signaling, the CagA/c-Met association was also observed in the absence of CD44 expression and signaling. Suzuki *et al*. [19] demonstrated that CagA CM motifs interact with Met leading to sustained PI3K-AKT signaling in response to *H*. *pylori*, leading to β-catenin activation and cellular proliferation. We advance our understanding of *H*. *pylori*-induced epithelial cell proliferation by demonstrating that CD44 acts as a coreceptor for c-Met in response to bacterial infection. The isoform containing exon v6 (CD44v6) acts as the coreceptor for c-Met in tumor cell lines [20,21]. The coreceptor function of CD44v6 for c-Met is of particular interest given that studies pinpoint CD44v6 as a marker of early invasive intramucosal gastric carcinoma [18]. Whether CD44v6 acted as a coreceptor for the function of c-Met in response to *H*. *pylori* infection was unknown. However, we found that *H*. *pylori*-induced proliferation was blocked in hFGOs pre-treated with the CD44v6 neutralizing antibody, and thus indicating that CD44v6 plays a functional role in *H*. *pylori-*induced proliferation in the human gastric epithelium.

Our data suggest a coreceptor function of CD44 for c-Met in response to *H*. *pylori* infection. The coreceptor function of CD44v6 for c-Met is shown to be dependent on HGF binding [20]. Indeed, we find that in the absence of CD44 expression and signaling HGF does not coimmunoprecipiate with c-Met and *H*. *pylori* fails to induce c-Met phosphorylation. In support of our findings, HGF binding to c-MET results in receptor homodimerization and phosphorylation of two tyrosine residues (Y1234 and Y1235) located within the catalytic loop of the tyrosine kinase domain [40], and subsequent, phosporylation of tyrosines 1349 and 1356 in the carboxy-terminal tail [41]. When these tyrosines become phosphorylated, they recruit signaling effectors that includes phosphatidylinositol 3-kinase (PI3K). When Y1313 is phosphorylated, it binds and activates PI3K, which promotes cell viability, motility and proliferation [42]. The extracellular domain of CD44v6 is necessary for c-Met activation, and this is dependent on HGF binding [20]. Indeed in the mFGOs, in response to *H*. *pylori* infection, HGF also coimmunoprecipitated with c-Met. The collaboration between c-Met and CD44v6 contributes to another bacterial infection, for example the invasion of *Listeria monocytogenes* into target cells [43]. Based on evidence demonstrating a functional role of CD44 in bacterial infection [43] including *H*. *pylori* infection, we conclude that the activation of c-Met is not only dependent on binding of *H*. *pylori* but in addition requires adhesion molecule CD44v6 as a co-receptor. In addition, although the coreceptor function of CD44v6 for c-Met is shown to be dependent on HGF binding, we show here for the first time that this signaling pathway mediates *H*. *pylori*-induced epithelial cell proliferation.

In the well-established Mongolian gerbil model of gastric cancer [35,36], animals treated with CD44 peptide inhibitor Pep1, resulted in the inhibition of *H*. *pylori-*induced proliferation and associated atrophic gastritis. Consistent with previous findings, infection with *H*. *pylori* for 6 weeks induced a significant inflammatory response, accompanied with the development of atrophic gastritis and metaplasia [35,44]. Using Pep1, a peptide that blocks the interaction between hyaluronic acid and CD44, we found that *H*. *pylori*-induced atrophic gastritis was inhibited. Importantly, infected gerbils that received Pep1 exhibited a significant reduction in actively proliferating cells. In support of our studies, a previous report by Khurana *et al. [39]* found that CD44 is a key coordinator of cell proliferation in a model of chemically-induced parietal cell atrophy, through downstream activation of STAT3. These findings strongly suggest that future therapeutic targets could include CD44 inhibition, to prevent *H*. *pylori*-induced hyperproliferation and cancer progression.

Our knowledge of *H*. *pylori* pathogenesis is predominantly based on data generated from gastric cancer cell lines or *in vivo* animal models of inflammation. Thus, limitation in acquiring such knowledge has been attributed to the inability to evaluate molecular mechanisms of bacterial and host cell interactions in a setting of a sustained gastric epithelial cell diversity and polarity. Our current work reports the development and use of a model of primary human and mouse cultured gastric epithelial cells. These models recapitulate key features of the gastric environment including the presence of the major gastric cell lineages and a polarized epithelium. In particular, we and others [25] report that the hFGO culture system represents a vital new technique for modeling *H*. *pylori* infection within the normal human tissue *in vitro*. As reported in similar mouse- and human-derived gastric organoid cultures [22,24,29], our study takes advantage of the presence of a defined lumen in these models, allowing us to inject live *H*. *pylori* directly into the gastric organoid and assay the epithelial response without the influence of host recruited factors. This contribution is significant because it provides knowledge required to potentially develop techniques to disrupt bacterial colonization and prevent disease progression. Our study helps to uncover a potential mechanism of *H*. *pylori*-induced proliferation in rodent and human tissue, using both *in vivo* established models of *H*. *pylori* infection and gastric cancer, as well as a novel epithelial cell culture system.

## Materials and Methods

### Ethics Statement

All mouse studies were approved by the University of Cincinnati Institutional Animal Care and Use Committee (IACUC) that maintains an American Association of Assessment and Accreditation of Laboratory Animal Care (AAALAC) facility. Human fundus was collected during sleeve gastrectomies (IRB protocol number: 2013–2251).

### *H*. *pylori* culture

*Helicobacter pylori* (*H*. *pylori*) strain LSH100, a descendant of the clinical isolate G27 [30], G27 wild type [31] (CagA^*+*^), a mutant G27 strain bearing a *cagA* deletion (Δ*cagA*::*cat*) [45] and *H*. *pylori* 7.13 strain were grown on blood agar plates containing Columbia Agar Base (Fisher Scientific), 5% horse blood (Colorado Serum Company), 5 µg/ml vancomycin and 10 µg/ml trimethoprim as previously described [46]. Plates were incubated for 2–3 days at 37°C in a humidified microaerophilic chamber [47].

### *H*. *pylori* animal inoculations

Bacteria were harvested and resuspended in filtered *Brucella* broth (BD biosciences) supplemented with 10% fetal calf serum. After 12 hours of growth at 37°C in a humidified microaerophilic chamber, bacteria were harvested, resuspended in filtered *Brucella* broth and C57/BL6 mice (The Jackson Laboratory, stock number: 000664), B6.129(Cg)-Cd44tm1Hbg/J (CD44-defiecient) mice (The Jackson Laboratory, stock number stock number: 005085) or Mongolian gerbils (Charles River) were inoculated by oral gavage with 10^8^ bacteria per 200 µl of *Brucella* broth. Mice were infected with either G27 wild type or a mutant G27 strain bearing a *cagA* deletion (Δ*cagA*::*cat*). Mongolian gerbils were inoculated with *H*. *pylori* strain 7.13. Uninfected control mice were administered 200 µl of *Brucella* broth. Mice were analyzed 4 weeks post-inoculation. Mongolian gerbils were analyzed 6 weeks post-inoculation.

*H*. *pylori* colonization was quantified using the culture method previously published [47]. Briefly, the wet weight of gastric tissue collected from uninfected and infected animals was measured. Tissue was homogenized in 1 ml saline and diluted 1/100 and spread on blood agar plates containing *Campylobacter* Base Agar (Fischer Scientific), 5% horse blood (BD Diagnostic Systems), 5µg/ml vancomycin and 10µg/ml trimethoprim. Plates were incubated for 7–10 days at 37^o^C in a humidified microaerophilic chamber. Single colonies from these plates tested positive for urease (BD Diagnostic Systems), catalase (using 3% H_2_O_2_) and oxidase (DrySlide, BD Diagnostic Systems). Colonies were counted and data normalized using the tissue wet weight and expressed and colony forming units (CFU)/g tissue.

### Hyaluronan (HA) blocking peptide

Specific hyaluronan (HA) blocking peptide Pep 1 (NH2-GAHHWQFNALTVRGGGS-CONH2) and scrambled control (NH2-WRHGFALTAVNQGGGS-CONH2) [48] peptides were synthesized by Pierce Biotechnology (Thermo Scientific-3747, Rockford, IL). Pep1 and control peptides were administered by intraperitoneal injection to Mongolian gerbils at a concentration of 10 mg/kg of body weight three times a week for 6 weeks after *H*. *pylori* infection.

### Histological scoring

Mongolian Gerbils were euthanized 6 weeks post-infection, stomachs were divided longitudinally for representative sections from both sides of the tissue and stained for Hematoxylin & Eosin (H&E). H&E stains were analyzed by using the updated Sydney classification system for histological scoring of gastritis [49].

### Immunohistochemical staining

Mice and Mongolian gerbils were injected with BrdU (300 mg/kg) 24 hours prior to analysis. Stomach sections spanning both the fundic and antral regions collected from experimental animals were fixed for 16 hours in Carnoy’s Fixative, paraffin embedded and sectioned at 5 µM. Prepared slides were deparaffinized with antigen retrieval performed by submerging in boiling solution (1:100 dilution Antigen Unmasking Solution in dH_2_O, Vector Laboratories, H-3300) for 10 minutes followed by 20 minutes at room temperature. Sections were then blocked with 5% BSA/PBS for 20 minutes at room temperature. BrdU color development was performed according to manufacturer’s protocol (Roche, Cat. No. 11 296 736 001). Immunohistochemical slides were dehydrated and mounted using Permount and images viewed and captured under light microscopy (Olympus BX60 with Diagnostic Instruments “Spot” Camera).

### Development and culture of mouse-derived fundic gastric organoids (mFGOs)

Gastric fundic organoids were prepared based on our recently reported protocol [22,50]. Primary epithelial cells from adult stomach tissue were cultured as 3-dimensional structures called mouse-derived fundic gastric organoids (mFGOs). Stomachs were dissected from mice along the greater curvature and washed in ice-cold Ca^2+^/Mg^2+^-free Dulbecco’s Phosphate Buffered Saline (DPBS). The stomach was stripped from muscle and visible blood vessels. Gastric fundus was further separated and cut into approximately 5 mm^2^ pieces. Tissue was then incubated in 5 ml of 5 mM EDTA for 2 hours at 4°C with gentle shaking. The EDTA was replaced with 5 ml chelation buffer (1 g D-sorbital, 1.47 g sucrose in 100 ml DPBS). Next tissue was shaken vigorously for approximately 2 minutes to dissociate glands. Dissociated glands were centrifuged at 150 g for 5 minutes then were embedded in *Matrigel* (BD Biosciences) supplemented with Advanced DMEM/F12 medium (Invitrogen), Wnt conditioned medium [22], R-spondin conditioned medium [22] supplemented with gastric growth factors including bone morphogenetic protein inhibitor, Noggin (PeproTech), Gastrin (Sigma), Epidermal grow factor (EGF, PeproTech) and Fibroblast growth factor 10 (FGF-10, PeproTech) as previously described [22,50]. Glands grew into organoids by 1–2 days. After 4 days in culture mFGOs were cultured on the inner well of polyester Transwell inserts (0.4 µm pore size, catalogue number 3460, Corning Lifescience), while immortalized stomach mesenchymal cells (ISMCs) were cultured in the base of the chamber of the Transwell according to our published protocol [22]. Organoids were co-cultured for a further 3 days prior to *H*. *pylori* infection and treatments.

### Development and culture of human-derived fundic gastric organoids (hFGOs)

Human fundic gastric organoids (hFGOs) were generated independently of the recently reported protocol [25]. The fundic mucosa was stripped away from the muscle layer, and then cut into 5 mm^2^ pieces and washed 3 times in sterile DPBS without Ca^2+^ and Mg^2+^. The mucosa was transferred to DMEM/F12 (catalogue number 1263–010, Gibco Life Technologies) supplemented with 10mM HEPES, 1%Penicillin/Streptomycin and 1X Glutamax, and incubated while stirring and oxygenated in a 37^o^C water bath with Collagenase (from *Clostridium histolyticum*, Sigma C9891, 1 mg/ml) and bovine serum albumin (2 mg/ml) to release glands from the tissue. After 15–30 minutes of incubation collected glands were washed in sterile phosphate buffered saline with Kanamycin (50 mg/ml) and Amphotericin B (0.25 mg/ml)/Gentamicin (10 mg/ml), centrifuged at 200 xg, resuspended in the appropriate volume of Matrigel (50 µl of Matrigel/well), and subsequently cultured in human gastric organoid media (DMEM/F12 supplemented with 10mM HEPES, 1X Glutamax, 1% Pen/Strep, 1X N2, 1X B27, 1mM N-Acetylcystine, 10mM Nicotidamide, 50ng/mL EGF, 100ng/mL Noggin, 20% R-Spondin Conditioned Media, 50% Wnt Conditioned Media, 200ng/mL FGF10, 1nM Gastrin, 10uM Y-27632, Kanamycin (50 mg/ml) and Amphotericin B (0.25 mg/ml)/Gentamicin (10 mg/ml)) (Table 1). Glands grew into organoids by 7 days at which time hFGOs were *H*. *pylori* infected and treated. We did not observe significant variations in organoid growth between donor gastric glands.

**Table 1:** Culture media for human-derived fundic gastric organoids (hFGOs).

Reagent	Supplier	Cat No.	Stock Conc.	Final Concentration
Glutmax	Invitrogen	35050–079	100X	1X
Penicillin/Streptomycin	Invitrogen	15140–122	100X	1X
N2 Supplements	Invitrogen	17502–048	100X	1X
B27 Supplements	Invitrogen	17504–044	50X	1X
N-Acetylcysteine	Sigma-Aldrich	A9165–5G	500mM	1mM
Nicotidamide	Sigma-Aldrich	N0636	1M	10mM
EGF	PeproTech	AF-100–15	500ug/ml	50ng/ml
Noggin	PeproTech	25038	100ug/ml	100ng/ml
FGF10	PeproTech	100–26	100ug/ml	200ng/ml
Gastin1	Tocris	3006	10uM	1nm
Y-27632	Sigma-Aldrich	Y0503	10mM	10uM
ADMEM/F12	Invitrogen	12634–010	-	30%
R-spondin Conditioned Media	In house			20%
WNT Conditioned Media	In house			50%
Amphotericin/Gentamicin	Gibco	50–064	100X	1X
Kanamycin	A.G. Scientific	K-1022	100X	1X

### Gastric organoid microinjections and treatments

*H*. *pylori* strains G27 and ΔCagA were grown on blood agar plates as described above, and prior to injection, a group of organoids were pre-treated for one hour with a highly selective, high affinity c-Met inhibitor (100 µg/ml, PF04217903 mesylate, Tocris Bioscience Cat.# 4239). CD44-deficient mouse-derived organoids were treated with Wnt agonist (1µM CALBIOCHEM, catalogue number 681665). The hFGOs were pretreated with CD44v6 antibody 1 hour prior to *H*. *pylori* infection at a concentration of 100 ng/ml. Organoids (mFGOs and hFGOs) cultured for 7 days were injected with 200 nl of *Brucella* broth using a Nanoject II (Drummond) microinjector, such that each organoid received approximately 2x10^5^ bacteria. Twenty-four hours after injection, organoids were harvested by washing in ice-cold DPBS to remove Matrigel, followed by either RNA isolation by TRIzol, protein isolation by M-PER Mammalian Protein Extraction Reagent (Thermo Scientific, IL) or EdU labeling.

### Immunofluorescence

The EdU solution was added to the organoid medium of either mFGOs or hFGOs for uptake for 1 hour. EdU staining was performed using the Click-iT Alexa Fluor 594 Imaging Kit, according to the manufacturer’s instructions (Life Technologies). The mFGOs were fixed with 4% formaldehyde for 20 minutes, followed by permeabilization with 0.5% Triton X-100 in DPBS for 20 minutes at room temperature. Blocking was done with 2% normal goat serum for 20 minutes at room temperature. The organoids were then incubated at 4^o^C overnight with an antibody specific for E-cadherin (Santa Cruz Biotechnology sc-59778, 1:100). Following this, organoids were incubated with Alexa Fluor 488 again overnight at 4^o^C. After incubation with the secondary antibodies organoids were counterstained using nuclear stain (Hoechst 33342, 10 µg/ml, Invitrogen) for 20 minutes at room temperature. Organoids were visualized using the Zeiss LSM710.

### Immunoprecipitation and Western blot analysis

Both mFGOs and hFGOs were harvested from Matrigel using ice-cold DPBS without Ca^2+^/Mg^2+^ and lysed in M-PER Mammalian Protein Extraction Reagent (Thermo Scientific, IL) supplemented with protease inhibitors (Roche) according to the manufacturer’s protocol. For the immunoprecipitation, c-Met antibody (AbCam ab59884) was added to the cell lysates (1:50 dilution) overnight at 4^o^C. Protein A/G Plus Agarose beads (Santa Cruz sc-2003) were washed with PSB and added to the cell lysate mixture overnight at 4^o^C. Cell lysate mixtures were resuspended in 40 µl Laemmli Loading Buffer containing beta-mercaptoethanol (Bio-Rad Laboratories, CA) before western blot analysis. Samples were loaded onto 4–12% Tris-Glycine Gradient Gels (Invitrogen) and run at 80 V, 3.5 hours before transfer to nitrocellulose membranes (Whatman Protran, 0.45 µM) at 105 V, 1.5 hours at 4^o^C. Membranes were blocked for 1 hour at room temperature using KPL Detector Block Solution (Kirkegaard & Perry Laboratories, Inc.). Membranes were incubated for 16 hours at 4°C with a 1:2000 dilution of either anti-GAPDH (Millipore, MAB374), 1:100 dilution of anti-phospho Tyrosine (Santa Cruz sc-7020), anti CagA (Abcam ab90490), anti-CD44 (Abcam ab51037), anti c-Met (Abcam ab59884), or anti-HGF (Abcam ab83760) followed by a 1 hour incubation with a 1:1000 dilution anti-mouse or anti-rabbit Alexa Fluor 680 (Invitrogen). Blots were imaged using a scanning densitometer along with analysis software (Odyssey Infrared Imaging Software System).

### Quantitative RT-PCR

Total RNA was isolated from mFGOs, hFGOs and gastric glands using TRIzol (Life Technologies) according to manufacture’s protocol. A High Capacity cDNA Reverse Transcription Kit synthesized cDNA from 100 ng of RNA following protocol provided by Applied Biosystems. Real-time PCR assays were utilized for the following genes in the mouse-derived organoids: GAPDH, alpha-Smooth Muscle Actin (Mm00725412_s1), Zeb1 (Mm00495564_m1) and 2 (Mm0049713_m1), TWIST 1 (Mm04208233_g1) and 2 (Mm000495564_m1), SNAIL 1 (Mm01249564_g1) and 2 (Mm00441531_m1). Cell lineage markers were determined by RT-PCR for HK-ATPase (Hs01026288_m1), gastrin (Hs01107047_m1), Muc5ac (Hs00873651_mH), and Muc6 (Hs01674026_g1). PCR amplifications were done with pre-validated 20X TaqMan Expression Assay primers, 2X TaqMan Universal Master Mix (Applied Biosystems), and cDNA template, in a total volume of 20 µL. Amplifications were performed with duplicate wells in a StepOne Real-Time PCR System (Applied Biosytems), and fold change was calculated at (Ct-Ct high) = n target, 2ntarget/2nGAPDH = fold change where Ct = threshold cycle.

### Flow cytometric analysis of cell lineages within hFGOs

The hFGOs were washed with cold DPBS without Ca^2+^/Mg^2+^ to remove Matrigel, and suspended in 2 ml Accutase (Innovative Cell Technologies, Cat.# AT-104) for 5 minutes at 37^o^C. Organoids were broken into single cells using an 18G needle, passed through 20 times. Single cells were fixed and permeablized using the FIX&PERM Kit from Invitrogen (catalogue number GAS004), according to manufacturer’s instructions. A first tube of cells was co-stained with lectin FITC labeled *Ulex europaeus* (UEAI, Sigma Aldrich), lectin *Griffonia simplicifolia* Alexa Fluor 647 *(*GSII, Molecular Probes) and then rabbit anti-pepsinogen C (Abcam, ab104595) followed by an anti-rabbit IgG PE secondary antibody (Abcam, ab7070), all at a 1:100 dilution. A second tube of cells was co-stained for chromogranin A antibody (Abcam, ab15160) followed by an anti-rabbit IgG PE conjugated secondary antibody, and anti-HK-ATPase (MA3–923, Affinity Bioreagents) followed by an anti-mouse IgG FITC conjugated (Abcam, ab6785), all at a 1:100 dilution. The antibody incubations were done for a period of 20 minutes at room temperature. The stained cells were analyzed using the FACSCalibur flow cytometer (BD Biosciences) followed by the FloJo software (Tree Star, Ashland, OR).

### Statistical analyses

The significance of the results was tested by two-way ANOVA using commercially available software (GraphPad Prism, GraphPad Software, San Diego, CA). A P value <0.05 was considered significant.
